# Lower chronic neck-back with higher planetary health diet adherence

**DOI:** 10.3389/fnut.2026.1855662

**Published:** 2026-07-10

**Authors:** Linfeng Wang, Libao Zhang, Xiaojun Yu, Changhui Xue, Chengwu Lu, Xuming Wang, Hong Ye

**Affiliations:** 1 Department of Orthopedic Surgery, Nanping First Hospital Affiliated to Fujian Medical University, Nanping, China; 2 Minbei Orthopedic Research Institute, Nanping, Fujian Province, China; 3 Department of Spine Surgery, Honghui Hospital, Xi‘an Jiaotong University, Xi'an, Shaanxi Province, China; 4 Shaanxi Key Laboratory of Spine Bionic Treatment, Xi'an, Shaanxi, China; 5 Department of Orthopedics, Jilin Province People's Hospital, Changchun, Jilin Province, China

**Keywords:** back pain, chronic pain, dietary pattern, neck pain, planetary health diet, UK biobank

## Abstract

**Background:**

This study aimed to examine the association between adherence to the Planetary Health Diet (PHD) and the prevalence of chronic neck-back pain.

**Methods:**

This cross-sectional analysis used baseline data from 97,543 participants in the UK Biobank with complete dietary, outcome, and covariate data. The PHD score was constructed from up to five 24-h dietary recalls. Chronic neck-back pain was defined as self-reported neck/shoulder or back pain in the previous month lasting ≥3 months and interfering with daily activities. Multivariable logistic regression, restricted cubic splines, and subgroup analyses were performed.

**Results:**

Overall, 22.5% of participants reported chronic neck-back pain. In the primary analysis, each 10-point increase in the PHD score was associated with lower odds of prevalent chronic neck-back pain (OR = 0.99, 95% CI: 0.97–1.00). In participants aged < 60 years, the association was stronger (OR = 0.97, 95% CI: 0.96–0.99). The highest PHD quartile showed 7% lower odds of prevalent chronic neck-back pain compared with the lowest quartile (OR = 0.93, 95% CI: 0.88–0.98).Effect modification analyses revealed that the protective effect was most pronounced in women aged < 60 years (OR=0.96, 95% CI: 0.94–0.98). Sensitivity analyses using alternative outcome definitions (ICD-10 codes, single-site pain) and exposure thresholds confirmed the robustness of these findings.

**Conclusions:**

Higher adherence to the Planetary Health Diet was associated with lower odds of prevalent chronic neck-back pain, particularly among adults aged < 60 years, with an approximately linear dose-response pattern. These findings provide the first prospective evidence that a sustainable, plant-centric dietary pattern may be associated with a lower risk of chronic spinal pain. However, further validation in independent populations is needed before broad clinical application can be recommended.

## 1 Introduction

Chronic neck and back pain has become the leading musculoskeletal disorder contributing to the global burden of non-fatal diseases and loss of healthy life years (1, 2). Epidemiological data indicate that chronic neck and back pain maintains a persistently high prevalence rate among adults and shows an increasing trend of onset in younger populations, with individuals aged < 60 now constituting the most affected and high-risk group (3–5). The pathogenesis of this condition is complex, with traditional research primarily focusing on risk factors such as poor posture, physical workload, psychological stress, and spinal degenerative changes (6–8). However, dietary nutrition—a core dimension of lifestyle that is both modifiable and reversible—has yet to be fully explored and systematically validated in its potential association with chronic neck and back pain.

In the field of dietary pattern research, the analytical framework focusing on single nutrients or individual foods has limitations, making it difficult to reflect the comprehensive impact of actual dietary structures on health (9, 10). In recent years, the Planetary Health Diet (PHD), an innovative dietary model that balances human health and ecological sustainability, has gained global recognition. It primarily consists of plant-based foods such as whole grains, vegetables, fruits, legumes, and nuts, paired with high-quality protein, while restricting the intake of red meat, saturated fatty acids, and added sugars (11). It has been proven to reduce the risks of chronic non-communicable diseases such as obesity, diabetes, cardiovascular diseases, and certain cancers (12–14). However, current research on the health benefits of the PHD remains focused on metabolic and cardiovascular diseases, with limited population-based evidence has evaluated the association between the PHD and musculoskeletal pain outcomes (15). The strength of its association with chronic neck and back pain, dose-response relationship, and population heterogeneity remain unknown.

At the same time, the existing research on the relationship between diet and neck and back pain has obvious deficiencies: first, it is difficult to establish the causal time sequence relationship because of the cross-sectional design; Second, the standardized and sustainable overall dietary pattern score was not used, and the exposure assessment was lack of uniformity and comparability; Third, the effect modification of key factors such as age and gender was not paid attention to, and the target population; Fourth, the lack of high-quality epidemiological evidence with large samples, long follow-up and multi-dimensional covariate control led to inconsistent research conclusions and limited clinical transformation value. The UK Biobank provides a large population-based dataset with standardized assessment of diet, pain-related outcomes, and a wide range of covariates, enabling a comprehensive evaluation of the association between dietary patterns and chronic neck-back pain. Based on baseline data from the UK Biobank, this study aimed to examine the association between adherence to the PHD and prevalent chronic neck-back pain. We further evaluated potential effect modification by age, sex, BMI, and physical activity, assessed the dose-response relationship using restricted cubic splines, and performed multidimensional sensitivity analyses. In this study, the PHD model was applied to the risk factors of chronic neck and back pain for the first time, in order to provide high-quality epidemiological evidence for the nutritional prevention of chronic neck and back pain, non drug intervention and the formulation of sustainable healthy diet guidelines, and provide a new scientific basis for the precise prevention and control of neck and back pain in young and middle-aged people.

## 2 Materials and methods

### 2.1 Research design and data sources

This study is based on the UK Biobank, which is a large prospective cohort study in the UK. From 2006 to 2010, more than 500000 adults aged between 37 and 73 were recruited in various assessment centers across the UK. The cohort systematically and comprehensively collected the subjects' sociodemographic characteristics, lifestyle, dietary intake, physical measurement indicators, biological samples and related medical and death registration data. The analysis sample of this study is limited to subjects who have completed at least two Oxford WebQ 24-h dietary recall surveys and have complete information on outcome variables and key covariates. The UK Biobank has ethical approval from the North West Multi-center Research Ethics Committee. All participants provided informed consent at enrollment. The current analyses were conducted under UK Biobank Application Number 78619. In terms of analysis strategy, this study first evaluated the association between PHD score and chronic neck and back pain in the whole sample population; Then, based on the results of age interaction test, the population under 60 years old was taken as the core target population for key restrictive analysis, and on this basis, further gender stratification and detailed sensitivity analysis were carried out.

### 2.2 Research object

In this study, subjects were screened through strict acceptance and exclusion criteria. The inclusion criteria included: (1) having WebQ dietary evaluation data that can be used to construct PHD score; (2) Complete at least 2 valid WebQ surveys; (3) Have clear information on the outcome of chronic neck and back pain; (4) The information of key covariates is complete.

Those who meet any of the following conditions will be excluded: (1) PHD score data is missing; (2) The outcome data (chronic neck and back pain) were missing; (3) Age, gender, education level, family income, BMI, physical activity level, smoking status, drinking status, diabetes history, total energy intake and other key covariates are missing.

After screening, a total of 97,543 subjects were included in the final sample, including 59994 subjects under 60 years old.

### 2.3 Dietary assessment and PHD score construction

The subjects' dietary exposure data were obtained from Oxford WebQ 24-h dietary review system of UK Biobank. In order to reduce the random error of single measurement and improve the representativeness of dietary exposure assessment, the average value of multiple WebQ measurements of each subject was used as the final exposure level. In order to ensure the accuracy of food intake estimation, only the food items with clear proportion size correspondence in UK biobank/WebQ system are included in the analysis.

The construction of PHD score refers to the previously published PHD scoring standards, covering 14 components: whole grains, potatoes, vegetables, fruits, dairy products, red meat, poultry, eggs, fish, beans and nuts, saturated fatty acids, unsaturated fatty acids and added sugar. According to the characteristics of each component, the scoring rules are divided into two dimensions: “moderate intake” (standard score) and “limited intake” (not exceeding the standard score), and the sum of the scores is the total score of PHD. The higher the total score, the more the subjects' overall dietary pattern complied with the recommended standard of PHD. In the continuous variable analysis, in order to enhance the clinical interpretability of the statistical results, the model was parameterized for every 10 points increase in PHD score; At the same time, this study also transformed PHD score into quartile classification variable and standardized variable (Z-score) to assist supplementary analysis. The specific food items corresponding to each component are shown in Supplementary Table S1.

### 2.4 Definition of outcome

The main outcome of this study was defined as chronic neck/back pain. The definition of symptomatology is based on the self-report of pain related problems in the baseline survey, which is constructed in two steps:

Assess whether the subjects have neck/shoulder pain and/or back pain that interferes with daily activities in the past 1 month. For the subjects who reported pain in the above parts, further confirm whether the duration of the pain symptom is ≥3 months.

The outcome criteria were as follows: patients without pain at corresponding sites in the past 1 month were defined as non cases (assigned a value of 0); Patients with pain in the past 1 month and duration ≥3 months were defined as cases (assigned a value of 1). Those who met the criteria for chronic neck/shoulder pain or chronic back pain were classified as cases of chronic neck-back pain. In addition, this study used the international classification of Diseases 10th Edition (ICD-10) code to construct the outcome index based on clinical diagnosis, which was specially used for sensitivity analysis. Relevant ICD-10 codes included (including M47.82, M48.02, M50.0, M50.3, M54.12, M47.86, M51.3, M54.5, M54.50, M54.56, M54.9). It mainly covers cervical degenerative diseases, intervertebral disc related diseases and cervical back idiopathic pain diseases.

### 2.5 Definition of covariates

The multivariable model included the following covariates for hybrid control:

Demographic sociological characteristics: age (continuous variable; broken down into < 60/ ≥60 years old, < 65/ ≥65 years old, or < 50, 50–59, 60–69, ≥70 years old groups in hierarchical analysis), gender (male, female), ethnicity (white, Asian, black and others), education level (University and above, A-level, secondary, vocal, none/other) and total household income (< 31k, 31k−51999, ≥52K pounds).

Lifestyle and physical characteristics: BMI (continuous variable in the main model; In effect modification analysis, they were classified as < 25.0, 25.0–29.9 and ≥30.0 kg/m^2^), physical activity (high, medium and low intensity according to IPAQ), smoking status (never, past, current), and drinking status (current, never, past).

Health status and energy intake: diabetes status (yes, no), total energy intake (continuous variable, kcal).

In the main analysis model, age, BMI and total energy intake were adjusted in the form of continuous variables, and the other covariates were included in the model in the form of classified variables.

### 2.6 Statistical analysis

First, the baseline characteristics of the subjects were displayed using descriptive statistical methods, and the characteristics were distributed according to the quartile of PHD score in the core population < 60 years old. Multivariate logistic regression model was used to evaluate the correlation between PHD score and chronic neck and back pain. Independent variables were included in the model in the form of continuous variables (every 10 points increased), quartile classification variables and quartile median (for linear trend test). The master model comprehensively adjusts age, gender, ethnicity, education, income BMI, physical activity, smoking, drinking, diabetes status and total energy intake. In the effect modification analysis, by introducing the product interaction term, the model focuses on the potential interaction of the following hierarchical factors: age, gender, BMI grouping and physical activity level grouping.

For the key population aged < 60 years, this study performed a multi-dimensional sensitivity analysis to verify the robustness of the results, including: (1) the use of alternative outcomes (chronic neck pain alone, chronic back pain alone, neck back pain in recent 1 month, neck back pain based on ICD coding); (2) Adjust the exposure parameterization mode (every 10 points increase, every 1 SD increase); (3) Increase the threshold of WebQ data inclusion (≥2 times, ≥3 times, ≥4 times); (4) Subjects with extreme energy intake were excluded; (5) Subjects with diabetes at baseline were excluded; (6) < 65 years old was used as the alternative age limit.

In addition, with the help of RMS macro package in R software, the dose-response relationship between PHD score and chronic neck and back pain in people under 60 years old was explored by using the restricted cubic splines (RCS) model. All statistical tests were bilateral tests, and *P* < 0.05 was identified as statistically significant. All the analysis is based on R software environment.

## 3 Results

### 3.1 Participant characteristics and full sample analysis

This study eventually included 97,543 effective subjects, and a total of 21,921 cases of chronic neck and back pain were identified, with an overall prevalence of about 22.5%. In the age stratification, 13,410 cases (22.4%) were in the population under 60 years old (*n* = 59,994); There were 8,511 cases (22.7%) in the population ≥ 60 years old (*n* = 37549). According to gender, the prevalence rate of female subjects (23.5%, 12102/51478) was slightly higher than that of male subjects (21.3%, 9819/46065). In the key population under 60 years old, there were differences in the distribution of demographic sociological characteristics, lifestyle and energy intake among different PHD score quartiles (Table 1). In general, the subjects with higher scores of PHD score showed more healthy distribution characteristics in education, family income and a number of lifestyle indicators.

**Table 1:** PHD components and corresponding WebQ items used in this study.

Type^*^	Dietary components	Food items from the UKB 24-h dietary recall	Criteria for score distribution based on PHD (g/2,500 kcal)				
			Minimum score (0 points)	Proportional score (0–10 points)	Maximum score (10 points)	Proportional score (10–0 points)	score (10–0 points) minimum score (0 points)
M	Total grains	Porridge/sweetened breakfast cereal/plain breakfast cereal/bran cereal/whole-wheat cereal/other cereal/sliced bread/brown rice/couscous/other cooked grains			≤ 232	232–464	≥464
O	Tubers or starchy vegetables	Chips/french fries/boiled or baked potatoes/mashed potatoes/sweet potato	0	0–50	50	50–100	≥100
A	Vegetables	Mixed vegetables/vegetable pieces/coleslaw/side salad/avocado/green beans/beetroot/broccoli/ butternut squash/cabbage/kale/ carrots/cauliflower/ celery/courgette/ cucumber/garlic/ leeks/lettuce/ mushrooms/onion/ parsnip/peas/ sweet peppers/spinach/ sprouts/	0	0–300	≥300		
		sweetcorn/ fresh tomatoes/ cooked or tinned tomatoes/ turnip/swede/ watercress/other vegetable intake					
A	Fruits	Mixed fruit/apple/banana/ berries/cherries/ grapefruit/grapes/ mango/melon/orange/ satsuma/peach/nectarine /pear/pineapple/ plum/other fruits/ stewed/cooked fruit/ prunes/other dried fruit	0	0–200	≥200		
O	Dairy foods	Milk/yogurt/low fat hard cheese/hard cheese/soft cheese/blue cheese/low fat cheese spread/cheese spread/cottage cheese/feta cheese/mozzarella /goat cheese/other cheese	0	0–250	250	250–500	≥500
M	Red meat	Beef/pork/lamb/sausages/ bacon/ham			≤ 14	14–28	≥28
O	Poultry	Breaded or fried poultry/poultry	0	0–29	29	29–58	≥58
O	Eggs	Whole eggs/omelet/eggs in sandwiches/scotch egg/other egg dishes	0	0–13	13	13–25	≥25
A	Fish	Shellfish/prawns/lobster/ crab/tinned tuna/oily fish/breaded fish/battered fish/white fish/other fish intake	0	0–28	≥28		
A	Legumes	Baked beans/pulses/broad beans	0	0–75	≥75		
A	Nuts	Salted peanuts/unsalted peanuts/salted nuts/unsalted nuts	0	0–50	≥50		
M	Saturated fats	Saturated fatty acids			0	0–11.8	≥11.8
A	Unsaturated fats	Monounsaturated fatty acids + *n-*3 fatty acids + *n*-6 fatty acids	0	0–40	≥40		
M	Added sugars	Free sugars			0	0–31	≥31

### 3.2 Main analysis results of the whole sample and people under 60 years old

**Figure 1:**
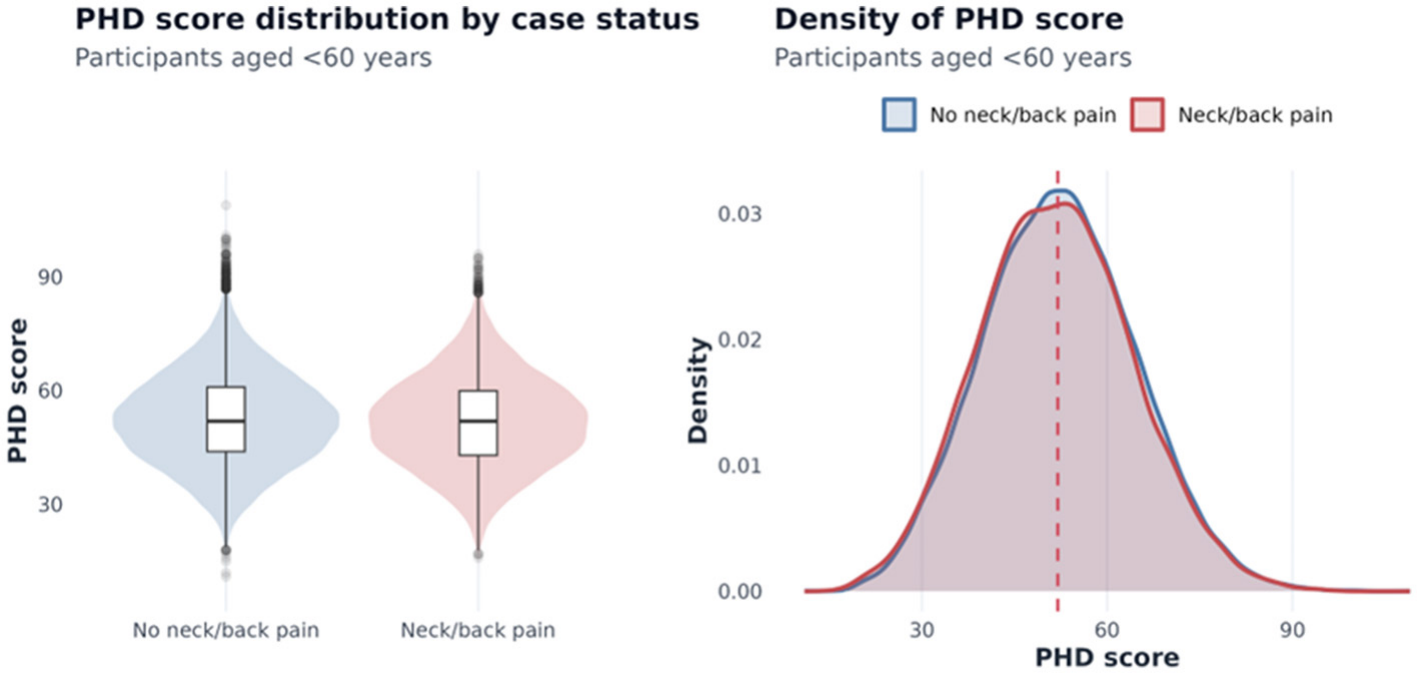
Distribution of PHD score by case status in participants aged < 60 years.

Logistic regression model showed that in the whole sample population, every 10 points increase in PHD score had a marginal significant weak inverse association with the odds of prevalent chronic neck-back pain, and the odds ratio (OR) was 0.99 (95% CI: 0.97, 1.00). This suggests that at the general population level, the more the dietary pattern complies with the recommendation of PHD, odds of prevalent chronic neck-back pain may be slightly lower, but the association was modest (Table 2; Figure 1). However, in the core population limited to < 60 years old, the above inverse association was stronger. For every 10 points increase in PHD score, the odds of prevalent chronic neck-back pain were significantly lower by 3% (OR = 0.97, 95% CI: 0.96, 0.99) (Table 2; Figure 1). The analysis using PHD score as a categorical variable also confirmed that compared with the lowest quartile group, the higher quartile group had lower odds of prevalent chronic neck-back pain, and the linear trend test result was significant (P for trend < 0.05) (Table 2).

**Table 2:** Main associations between PHD score and neck/back pain.

Population	Analysis	Comparison	OR (95% CI)	*P* value
Overall	Continuous	PHD score (per 10-point increase)	0.99 (0.97, 1.00)	0.049
	Quartiles	Q2 vs. Q1	0.98 (0.94, 1.02)	0.400
	Quartiles	Q3 vs. Q1	0.98 (0.94, 1.02)	0.360
	Quartiles	Q4 vs. Q1	0.98 (0.93, 1.02)	0.264
	Trend	*P* for trend	0.99 (0.98, 1.01)	0.278
Participants aged < 60 years	Continuous	PHD score (per 10-point increase)	0.97 (0.96, 0.99)	0.002
	Quartiles	Q2 vs. Q1	0.97 (0.92, 1.03)	0.314
	Quartiles	Q3 vs. Q1	0.97 (0.91, 1.02)	0.209
	Quartiles	Q4 vs. Q1	0.93 (0.88, 0.98)	0.010
	Trend	*P* for trend	0.98 (0.96, 0.99)	0.012

Models are adjusted for age, sex, ethnicity, educational level, income, BMI, physical activity, smoking, alcohol consumption, diabetes, and total energy intake. Continuous analysis is based on a 10-point increase in the PHD score.

### 3.3 Effect modification and subgroup analysis

Effect modification analysis showed that age was a significant heterogeneity factor affecting the association (Figure 1). In the subgroup of < 60 years old, the OR of PHD score (every 10 points increased) was 0.97 (95% CI: 0.96, 0.99); However, in the 60–69 year old population, the effect was weakened and not statistically significant (OR = 1.01, 95% CI: 0.99, 1.03). Further detailed analysis of age levels revealed that the protective effect was the most stable in the 50–59 age group (OR = 0.97, 95% CI: 0.95, 0.99), and the < 50 age group also showed a consistent negative trend (OR = 0.98, 95% CI: 0.95, 1.00). It is worth noting that the association in the opposite direction was observed in the elderly ≥70 years old (OR = 1.37, 95% CI: 1.04, 1.82). In view of the potential impact of sample size and survivor bias in this age group, this finding should be regarded as an exploratory result and should be interpreted with caution (Supplementary Table S2, Supplementary Figure S1). The interaction analysis by gender in the population under 60 years old found that the protective effect of female group was more prominent. In young women, the or for every 10 points increase in PHD score decreased to 0.96 (95% CI: 0.94, 0.98), while in men of the same age group, the or was 0.99 (95% CI: 0.96, 1.01) (Figure 1, Supplementary Table S3).

In conclusion, the age interaction model provides strong evidence that the negative correlation between PHD score and chronic neck and back pain has obvious age heterogeneity, and its protective effect is mainly concentrated in younger groups (especially subjects < 60 years old and 50–59 years old) (Supplementary Tables S5, S6).

### 3.4 Dose response relationship in people under 60 years old

**Figure 2:**
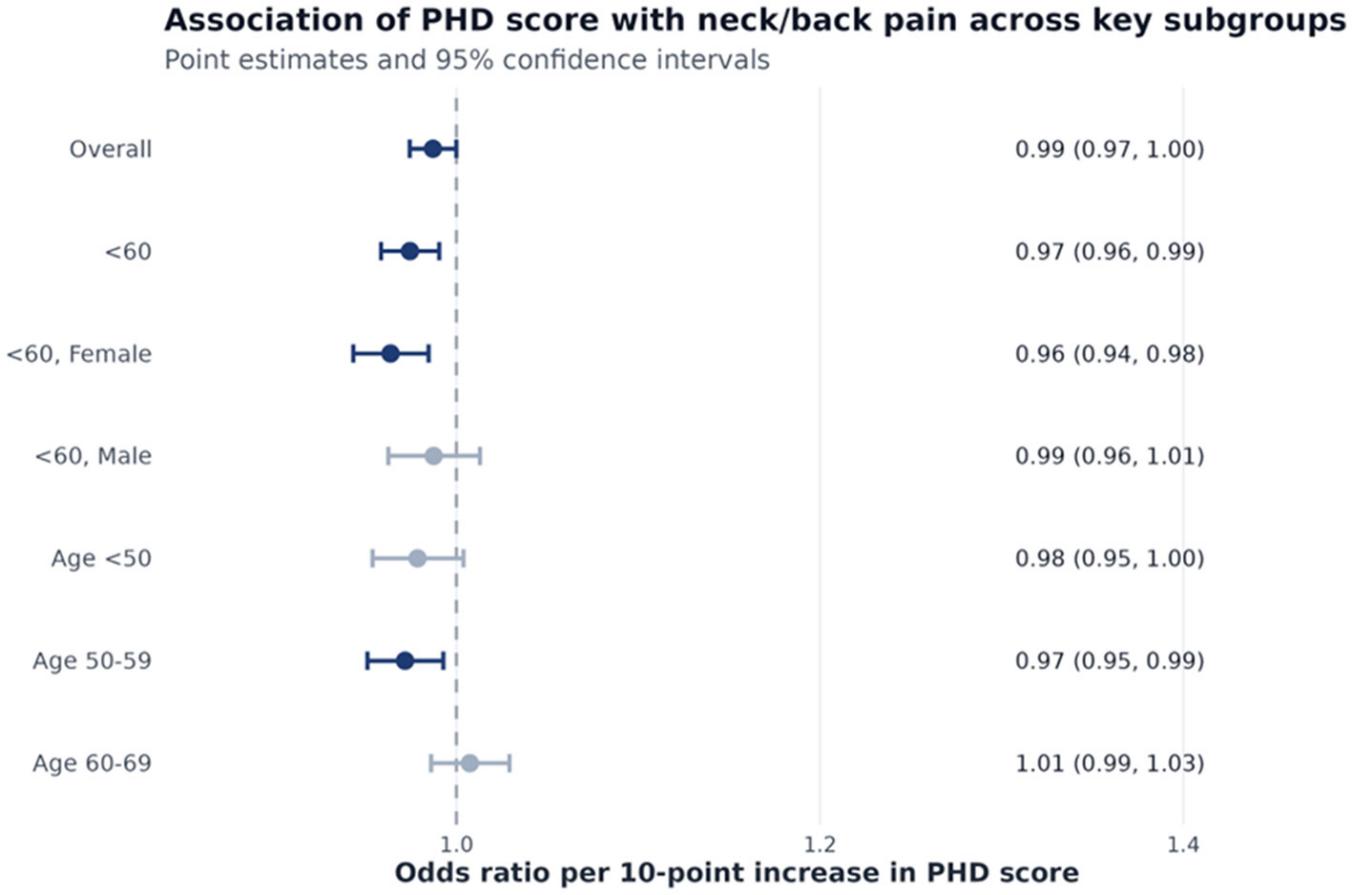
Association of PHD score with neck/back pain across key subgroups.

The continuous variable analysis based on the RCS showed that PHD score was approximately monotonically inversely associated with the odds of prevalent chronic neck-back pain in the population < 60 years old (Figure 2). With the steady rise of PHD score, the odds ratio of prevalent chronic neck-back pain continued to decline, and the protective trend was particularly obvious in the high and medium rating sections. Although the confidence intervals at both ends of the distribution were widened due to the sample size limit, no obvious nonlinear or reverse inflection point was observed in the overall fitting curve. This shows that the correlation between the two is more in line with the smooth near linear decrease in prevalence odds, rather than the threshold effect with strict boundaries.

### 3.5 Sensitivity analysis

**Figure 3:**
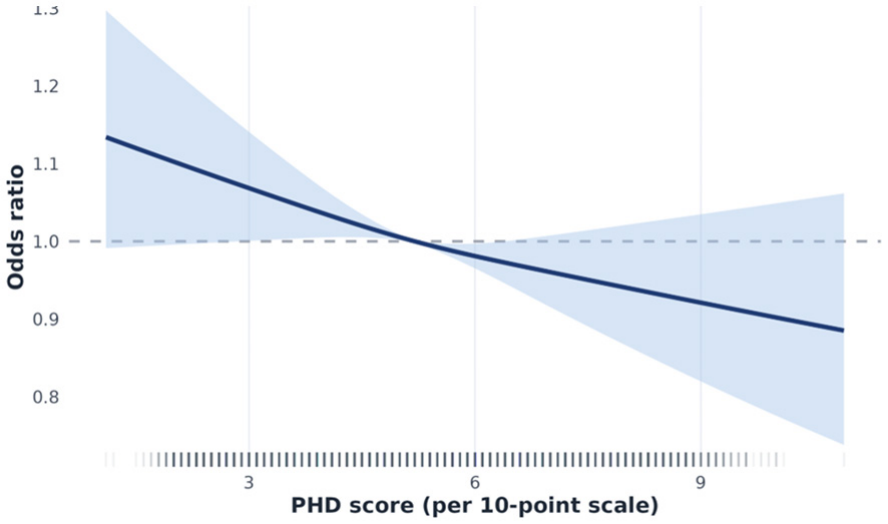
Dose-response association between PHD score and neck/back pain in participants aged < 60 years.

The results of multiple sensitivity analyses are highly consistent, further verifying the robustness of the main analysis (Figure 3, Supplementary Table S4, Supplementary Figure S2).

First of all, the sensitivity analysis of the outcome definition showed that the effect direction did not change when chronic neck/shoulder pain and chronic back pain were analyzed separately, when neck-back pain in the previous month was considered, or when the outcome was defined using ICD-10 clinical codes. In all analyses, higher PHD scores were consistently associated with lower pain risk. Because the number of cases with ICD coding diagnosis is relatively small, the confidence interval of point estimation under this outcome is wide, but the overall trend remains unchanged.

Secondly, in the robustness test of exposure parameters, the effect evaluation results are highly consistent whether it is modeled by every 10 points or every 1 SD. In addition, the severity of exposure assessment criteria was further improved (the threshold of WebQ completion times was increased from ≥2 times to ≥ 3 times and ≥ 4 times respectively), and the model estimation still maintained a negative correlation; Even if the statistical efficiency decreases slightly with the reduction of sample size (the confidence interval becomes wider), the core inference is still true. Finally, after excluding the subjects with extreme total energy intake, excluding the patients with baseline diabetes, and raising the cut-off point of age stratification to < 65 years old, the conclusions did not change substantially (Figure 3). The above comprehensive evidence shows that the exposure outcome association revealed in this study has excellent epidemiological reliability and robustness.

## 4 Discussion

Based on the UK Biobank large prospective cohort, this study systematically explored the association between the PHD model and prevalent chronic neck and back pain for the first time, and filled the research gap in the field of “overall dietary pattern musculoskeletal pain” through multi-dimensional subgroup analysis and dose-response relationship modeling. The core findings show that the adherence to PHD model was associated with lower odds of prevalent chronic neck and back pain, although the magnitude of the association in the overall population was relatively modest. This association appeared to be more evident in young and middle-aged people, people with low BMI and people with regular physical activity. This result not only responds to the previous epidemiological scientific hypothesis, but also provides a new dietary intervention target for the non-drug prevention strategy of chronic neck and back pain.

From the perspective of deep-seated mechanism, the protective effect of PHD may be related to three core paths: first, PHD is dominated by plant-based food, and the structure of restricting the intake of red meat and refined sugar may help reduce the level of chronic inflammation in the body(16), while inflammatory factors (such as IL-6 and TNF—α) have been reported to be involved in the process of spinal disc degeneration, cervical muscle aseptic inflammation and nerve endings sensitivity(17, 18), which could potentially contribute to pain perception and disease progression; Second, sufficient vegetables, fruits, nuts and beans in PHD provide rich magnesium, calcium, vitamin D and omega-3 fatty acids. These nutrients are the key material basis for maintaining spinal bone mineral density, muscle contraction function and nerve conduction efficiency (19–21), and might be associated with a lower risk of degenerative changes and pain; Third, previous studies have suggested that dietary patterns characterized by low saturated fat intake and high dietary fiber intake may be associated with better metabolic health and lower obesity risk (22–24). Since obesity is an important risk factor for neck and back pain (25), this may partially explain the stronger association observed among participants with lower BMI in the present study. However, the current study did not directly assess metabolic or biomechanical indicators, and therefore these potential mechanisms require further investigation. However, these potential mechanisms should be interpreted with caution, as the present study did not include inflammatory biomarkers or imaging indicators to directly verify these pathways. Future experimental and clinical studies are needed to confirm these hypotheses.

Combined with the current global public health policy and clinical practice orientation, the results of this study have important transformation and application value. In recent years, the World Health Organization (WHO) has incorporated musculoskeletal health into the comprehensive prevention and control framework for non communicable diseases (NCDs) (26, 27), and emphasized the core role of lifestyle intervention, including nutrition management and physical activity, in the prevention and control of chronic musculoskeletal diseases in its rehabilitation intervention guidelines (26); At the same time, more and more evidence shows that a sustainable diet model that takes into account health and the environment has protective significance for the maintenance of bone and muscle health(28); China's “Healthy China 2030” planning outline emphasizes the strategic direction of “prevention first”, and puts forward the policy requirements of giving full play to the unique advantages of traditional Chinese medicine and developing non drug therapy(29). Chronic pain has become an important public health problem in China (30), Under this policy framework, the promotion and application of non-drug and non-invasive interventions in the field of pain management have attracted increasing attention (29, 31). This study suggests that the PHD model not only conforms to the dietary concept of global sustainable development, but may be associated with a lower prevalence of chronic neck and back pain. However, given the modest effect size observed in the overall population, further studies are needed before these findings can directly inform clinical guidelines or population-level prevention strategies. At the same time, the “interaction of age, BMI and physical activity” found in this study can provide a basis for hierarchical intervention for different groups: for example, focus on promoting PHD mode to young and middle-aged people, and intervene the risk of neck and back pain in advance; For obese people, dietary guidance and weight management can be combined to simultaneously reduce the risk of neck and back pain and metabolic diseases, so as to achieve the public health benefits of “multiple diseases prevention”, which is in line with the current policy trend of comprehensive prevention and control of chronic diseases in the world.

The advantages of this study are mainly reflected in four aspects: first, relying on the prospective cohort design of UK Biobank with large samples and long follow-up, it effectively avoids the causal timing defects of previous cross-sectional studies, can clarify the prospective correlation between PHD mode and neck and back pain risk, and improves the causal inference strength of the research conclusion; Secondly, the standardized PHD score system was used to quantitatively evaluate the dietary structure, which solved the problems of inconsistent and poor comparability of dietary exposure assessment in previous studies, and ensured the reliability of the results; Thirdly, through multi-dimensional subgroup analysis and interaction test, the heterogeneity of the associated population was comprehensively revealed, and the core population of the protective effect was identified, which provided a scientific basis for precise intervention; Finally, the dose-response relationship between PHD score and the risk of neck and back pain was accurately described by using advanced statistical methods such as the restricted cubic spline model, which filled the blank in the quantitative research field of “dietary intake and disease risk”. However, there are still some limitations in this study: first, although the UK Biobank Cohort data is comprehensive, the study population is mainly white people in Europe, which lacks the verification of different ethnic groups in Asia and Africa, and there may be population stratification bias. In addition, the present study was conducted using a single database and did not include an independent external validation cohort. Therefore, the generalizability of our findings to other populations should be interpreted with caution. In the future, multi-ethnic and large sample cohort studies with external validation needed to be carried out to verify the extrapolation of the results; Second, dietary intake data were collected through 24-h review method and food frequency questionnaire. Although the quality was controlled, there may still be subject reporting bias, which can be further corrected in combination with biomarkers (such as serum nutrient levels); Third, this study only focused on the dietary pattern of PHD, and did not explore the specific association between different dietary components (such as nut intake, fish intake frequency) and neck and back pain. In the future, we can mine the core protective dietary components and refine the intervention targets through network analysis and other methods; Fourth, the study did not include intermediate outcomes such as neck muscle function and cervical imaging indicators, which made it difficult to fully explain the pathophysiological pathway of PHD mode affecting neck and back pain. In the follow-up, we can further improve the correlation mechanism chain by combining clinical imaging data and mechanism experiments.

In summary, this study identified an inverse association between adherence to the PHD model and chronic neck and back pain. Although the association in the overall population was statistically significant, its magnitude was relatively modest and should therefore be interpreted with caution. Its mechanism may be related to inflammation regulation, bone metabolism improvement and mechanical load reduction, although these potential pathways require further confirmation. Despite the above limitations, the prospective design, large sample advantage and multi-dimensional analysis of the study still provide high-quality evidence for the field. In the future, the application system of PHD diet mode in the prevention and control of chronic neck and back pain can be further improved through multi-ethnic validation, biomarker integration and mechanism in-depth exploration, providing scientific support for the transformation of chronic neck and back pain management mode from treatment to prevention, from individual intervention to population prevention and control.

## Data Availability

The original contributions presented in the study are included in the article/Supplementary material, further inquiries can be directed to the corresponding authors.
