# Supplementary material for: Lower chronic neck-back with higher planetary health diet adherence
Source: Front Nutr. 2026 Jul 10;13:1855662. doi: 10.3389/fnut.2026.1855662 (PMC13395737; doi:10.3389/fnut.2026.1855662)
Supplement: Supplementary file 1 [file Table_1.docx]

**Supplementary Table S1. Sex-stratified associations in participants aged <60 years**

| **Sex** | **Analysis** | **Comparison** | **OR (95% CI)** | **P value** |
| --- | --- | --- | --- | --- |
| Female | Continuous | PHD score (per 10-point increase) | 0.96 (0.94, 0.98) | <0.001 |
| Female | Quartiles | Q2 vs Q1 | 0.98 (0.91, 1.05) | 0.604 |
| Female | Quartiles | Q3 vs Q1 | 0.96 (0.89, 1.03) | 0.247 |
| Female | Quartiles | Q4 vs Q1 | 0.89 (0.83, 0.96) | 0.002 |
| Female | Trend | P for trend | 0.96 (0.94, 0.99) | 0.002 |
| Male | Continuous | PHD score (per 10-point increase) | 0.99 (0.96, 1.01) | 0.332 |
| Male | Quartiles | Q2 vs Q1 | 1.01 (0.93, 1.10) | 0.865 |
| Male | Quartiles | Q3 vs Q1 | 0.97 (0.89, 1.06) | 0.474 |
| Male | Quartiles | Q4 vs Q1 | 0.99 (0.91, 1.08) | 0.795 |
| Male | Trend | P for trend | 0.99 (0.97, 1.02) | 0.599 |

**Supplementary Table S2. Age-stratified associations**

| **Age group** | **Analysis** | **Comparison** | **OR (95% CI)** | **P value** |
| --- | --- | --- | --- | --- |
| <50 | Continuous | PHD score (per 10-point increase) | 0.98 (0.95, 1.00) | 0.096 |
| <50 | Quartiles | Q2 vs Q1 | 0.99 (0.91, 1.08) | 0.767 |
| <50 | Quartiles | Q3 vs Q1 | 0.96 (0.88, 1.05) | 0.342 |
| <50 | Quartiles | Q4 vs Q1 | 0.94 (0.86, 1.03) | 0.209 |
| <50 | Trend | P for trend | 0.98 (0.95, 1.01) | 0.164 |
| 50-59 | Continuous | PHD score (per 10-point increase) | 0.97 (0.95, 0.99) | 0.009 |
| 50-59 | Quartiles | Q2 vs Q1 | 0.95 (0.88, 1.02) | 0.154 |
| 50-59 | Quartiles | Q3 vs Q1 | 0.96 (0.89, 1.03) | 0.21 |
| 50-59 | Quartiles | Q4 vs Q1 | 0.92 (0.85, 0.98) | 0.018 |
| 50-59 | Trend | P for trend | 0.97 (0.95, 1.00) | 0.028 |
| 60-69 | Continuous | PHD score (per 10-point increase) | 1.01 (0.99, 1.03) | 0.501 |
| 60-69 | Quartiles | Q2 vs Q1 | 1.03 (0.96, 1.10) | 0.409 |
| 60-69 | Quartiles | Q3 vs Q1 | 1.04 (0.97, 1.11) | 0.294 |
| 60-69 | Quartiles | Q4 vs Q1 | 1.05 (0.98, 1.13) | 0.155 |
| 60-69 | Trend | P for trend | 1.02 (0.99, 1.04) | 0.156 |
| >=70 | Continuous | PHD score (per 10-point increase) | 1.37 (1.04, 1.82) | 0.029 |
| >=70 | Quartiles | Q2 vs Q1 | 0.88 (0.34, 2.25) | 0.787 |
| >=70 | Quartiles | Q3 vs Q1 | 1.45 (0.61, 3.51) | 0.401 |
| >=70 | Quartiles | Q4 vs Q1 | 2.66 (1.17, 6.30) | 0.022 |
| >=70 | Trend | P for trend | 1.42 (1.09, 1.88) | 0.011 |

**Supplementary Table S3. Interaction analyses**

| **Interaction** | **Model term** | **OR (95% CI)** | **P value** |
| --- | --- | --- | --- |
| PHD_score_10 × age_group2 | PHD_score_10:age_group2>=60 | 1.03 (1.01, 1.06) | 0.015 |
| PHD_score_10 × age_group2_65 | PHD_score_10:age_group2_65>=65 | 1.03 (1.00, 1.07) | 0.085 |
| PHD_score_10 × age_group4 | PHD_score_10:age_group450-59 | 0.99 (0.96, 1.02) | 0.618 |
| PHD_score_10 × age_group4 | PHD_score_10:age_group460-69 | 1.03 (0.99, 1.06) | 0.124 |
| PHD_score_10 × age_group4 | PHD_score_10:age_group4>=70 | 1.44 (1.12, 1.86) | 0.005 |
| PHD_score_10 × sex (overall) | PHD_score_10:sex2Male | 1.02 (0.99, 1.04) | 0.206 |
| PHD_score_10 × sex (<60) | PHD_score_10:sex2Male | 1.02 (0.99, 1.06) | 0.199 |
| PHD_score_10 × BMI group | PHD_score_10:bmi_group25-29.9 | 1.01 (0.98, 1.04) | 0.628 |
| PHD_score_10 × BMI group | PHD_score_10:bmi_group>=30 | 1.01 (0.97, 1.04) | 0.760 |
| PHD_score_10 × activity group | PHD_score_10:activity_group3moderate | 1.02 (0.99, 1.05) | 0.207 |
| PHD_score_10 × activity group | PHD_score_10:activity_group3low | 1.05 (1.02, 1.09) | 0.003 |

**Supplementary Table S4. Sensitivity analyses**

| **Analysis** | **Item** | **OR (95% CI)** | **P value** |
| --- | --- | --- | --- |
| Alternative outcomes | Chronic neck/shoulder pain (3+ months) | 0.98 (0.96, 1.00) | 0.038 |
| Alternative outcomes | Chronic back pain (3+ months) | 0.97 (0.95, 0.99) | 0.003 |
| Alternative outcomes | Neck/back pain last month | 0.98 (0.97, 1.00) | 0.021 |
| Alternative outcomes | ICD-coded neck/back pain | 0.99 (0.96, 1.03) | 0.754 |
| Exposure specification | Per 10-point increase | 0.97 (0.96, 0.99) | 0.002 |
| Exposure specification | Per 1-SD increase | 0.97 (0.95, 0.99) | 0.002 |
| WebQ repeat threshold | n_valid_instances >= 2 | 0.97 (0.96, 0.99) | 0.002 |
| WebQ repeat threshold | n_valid_instances >= 3 | 0.98 (0.96, 1.00) | 0.048 |
| WebQ repeat threshold | n_valid_instances >= 4 | 0.99 (0.96, 1.03) | 0.742 |
| Exclusion analysis | Exclude extreme energy intake (1st/99th percentile) | 0.98 (0.96, 0.99) | 0.003 |
| Exclusion analysis | Exclude participants with diabetes | 0.97 (0.96, 0.99) | 0.002 |
| Age-cut sensitivity | Participants aged <65 years | 0.98 (0.97, 1.00) | 0.018 |

**Supplementary Table S5. Stratified analyses by BMI group**

| **BMI group** | **Analysis** | **Comparison** | **OR (95% CI)** | **P value** |
| --- | --- | --- | --- | --- |
| <25 | Continuous | PHD score (per 10-point increase) | 0.99 (0.97, 1.01) | 0.22 |
| <25 | Quartiles | Q2 vs Q1 | 0.97 (0.91, 1.04) | 0.441 |
| <25 | Quartiles | Q3 vs Q1 | 0.97 (0.90, 1.04) | 0.437 |
| <25 | Quartiles | Q4 vs Q1 | 0.99 (0.92, 1.06) | 0.784 |
| <25 | Trend | P for trend | 1.00 (0.97, 1.02) | 0.793 |
| 25-29.9 | Continuous | PHD score (per 10-point increase) | 0.99 (0.97, 1.01) | 0.312 |
| 25-29.9 | Quartiles | Q2 vs Q1 | 0.98 (0.92, 1.05) | 0.629 |
| 25-29.9 | Quartiles | Q3 vs Q1 | 0.99 (0.93, 1.06) | 0.787 |
| 25-29.9 | Quartiles | Q4 vs Q1 | 0.98 (0.92, 1.05) | 0.617 |
| 25-29.9 | Trend | P for trend | 1.00 (0.97, 1.02) | 0.684 |
| >=30 | Continuous | PHD score (per 10-point increase) | 0.99 (0.96, 1.01) | 0.3 |
| >=30 | Quartiles | Q2 vs Q1 | 1.08 (0.99, 1.19) | 0.092 |
| >=30 | Quartiles | Q3 vs Q1 | 1.06 (0.97, 1.17) | 0.196 |
| >=30 | Quartiles | Q4 vs Q1 | 1.00 (0.91, 1.10) | 0.952 |
| >=30 | Trend | P for trend | 1.00 (0.97, 1.03) | 0.852 |

**Supplementary Table S6. Stratified analyses by physical activity group**

| **Physical activity** | **Analysis** | **Comparison** | **OR (95% CI)** | **P value** |
| --- | --- | --- | --- | --- |
| high | Continuous | PHD score (per 10-point increase) | 0.97 (0.95, 0.99) | 0.008 |
| high | Quartiles | Q2 vs Q1 | 0.95 (0.88, 1.02) | 0.134 |
| high | Quartiles | Q3 vs Q1 | 1.00 (0.93, 1.07) | 0.89 |
| high | Quartiles | Q4 vs Q1 | 0.91 (0.85, 0.98) | 0.016 |
| high | Trend | P for trend | 0.98 (0.96, 1.00) | 0.065 |
| moderate | Continuous | PHD score (per 10-point increase) | 0.99 (0.97, 1.01) | 0.173 |
| moderate | Quartiles | Q2 vs Q1 | 0.98 (0.92, 1.04) | 0.51 |
| moderate | Quartiles | Q3 vs Q1 | 0.94 (0.88, 1.00) | 0.058 |
| moderate | Quartiles | Q4 vs Q1 | 0.98 (0.92, 1.05) | 0.547 |
| moderate | Trend | P for trend | 0.99 (0.97, 1.01) | 0.338 |
| low | Continuous | PHD score (per 10-point increase) | 1.02 (0.99, 1.05) | 0.211 |
| low | Quartiles | Q2 vs Q1 | 1.10 (1.00, 1.22) | 0.052 |
| low | Quartiles | Q3 vs Q1 | 1.07 (0.97, 1.19) | 0.159 |
| low | Quartiles | Q4 vs Q1 | 1.10 (0.99, 1.21) | 0.068 |
| low | Trend | P for trend | 1.03 (0.99, 1.06) | 0.12 |


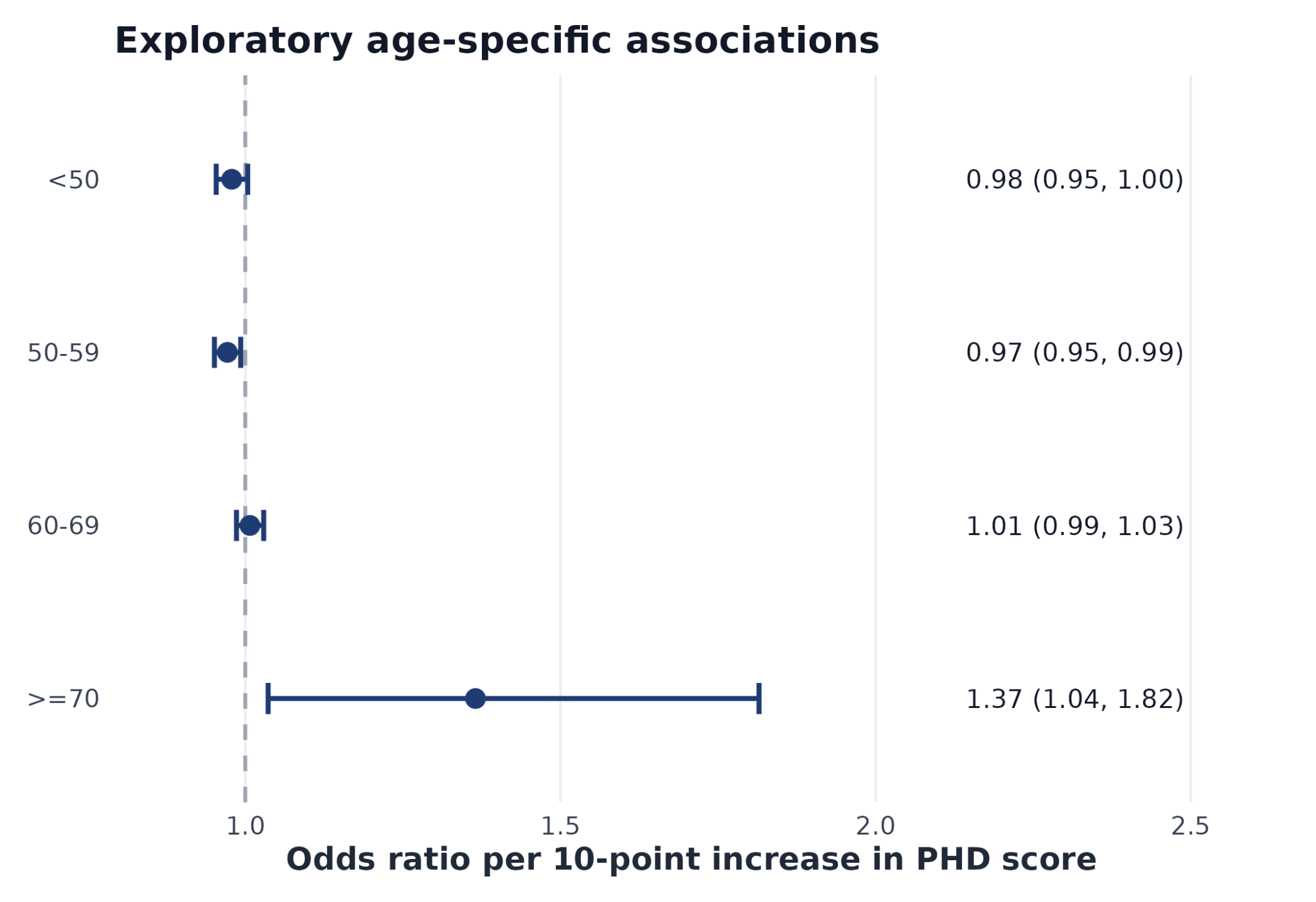


**Supplementary Figure S1. Exploratory age-specific associations**


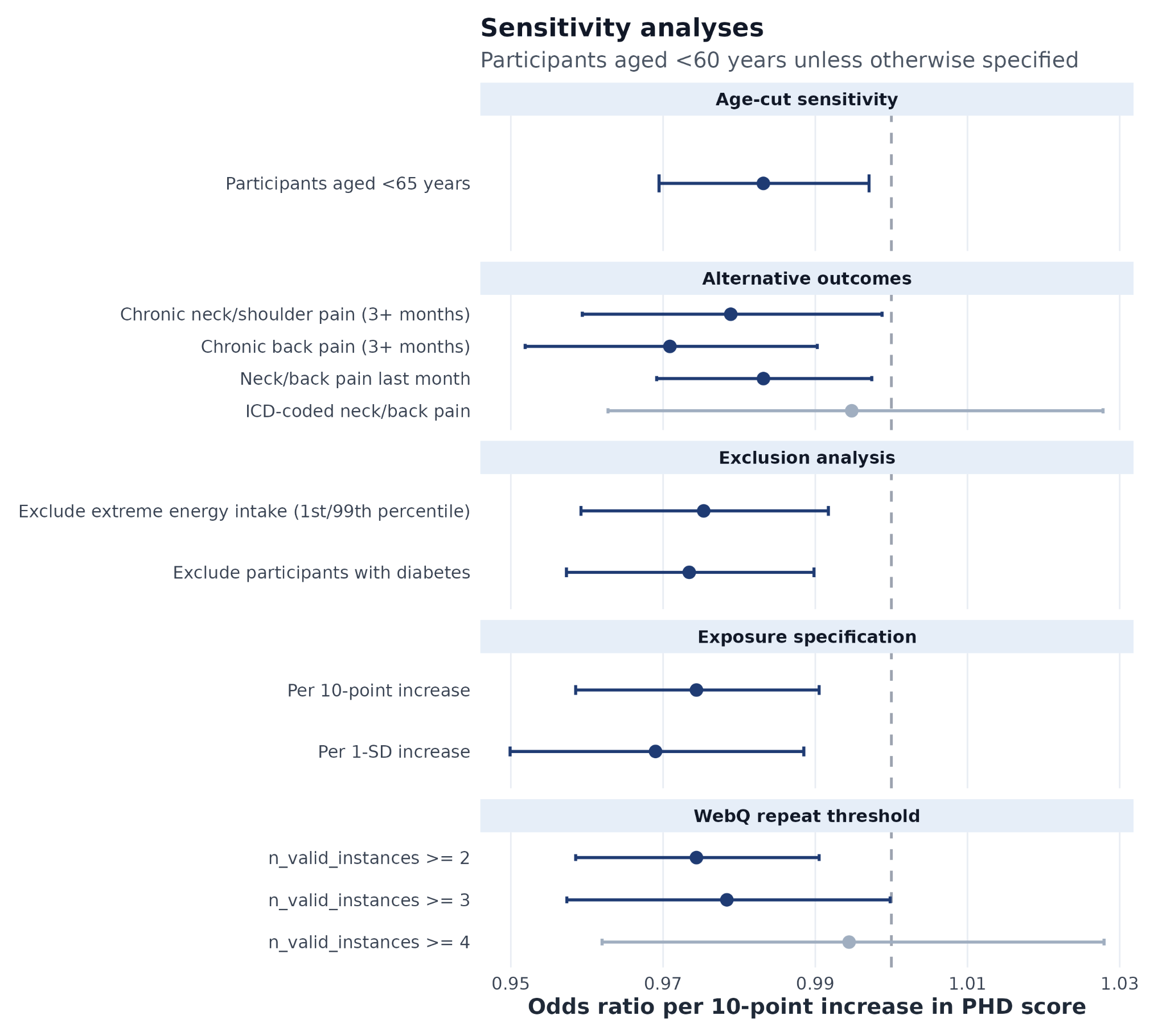


**Supplementary Figure S2. Sensitivity analyses**
